# Response comparison of PLC and SLC with magnetic resonance elastography after TACE

**DOI:** 10.1038/s41598-022-12478-w

**Published:** 2022-05-18

**Authors:** Y. Haas, M. P. Dosch, T. J. Vogl

**Affiliations:** University Hospital Frankfurt, Theodor-Stern-Kai 7, 60590 Frankfurt am Main, Germany

**Keywords:** Cancer, Medical research, Oncology, Physics

## Abstract

The aim of this study was to detect a response difference in primary (PLC) and secondary liver tumors (SLC) with magnetic resonance elastography (MRE) after TACE therapy. Thirty-one patients (25/31 male; mean age 69.6 years [range: 39–85 years]) with repeated TACE therapy of HCC were compared with twenty-seven patients (27/27 female; mean age 61.2 years [range 39–81 years]) with repeated TACE therapy of metastatic liver disease due to breast cancer. Both groups underwent either one (*n* = 31) or two (*n* = 27) repetitive magnetic resonance imaging (MRI) and MRE exams in 4- to 6-week intervals using a 1.5-T-scanner. MRE-based liver stiffness and size measurements were evaluated in tumorous lesions and in healthy liver lobe controls. PLC showed a significantly larger tumor size compared to SLC (26.4 cm^2^ vs. 11 cm^2^, *p* = 0.007) and a higher degree of stiffness (5.8 kPa vs. 5.1 kPa, *p* = 0.04). Both tumors decreased in size during the cycles (PLC: *p* = 0.8 and SLC: *p* < 0.0001) and lesions showed an increase in stiffness (PLC: *p* = 0.002 and SLC: *p* = 0.006). MRE demonstrates that PLC and SLC have similar responses to TACE therapy. PLC had a greater increase in stiffness and SLC got smaller. An increasing stiffness and decrease in size could show a good response.

## Introduction

Liver cancer can be classified in primary (abbr. PLC) and secondary tumors (abbr. SLC). The most common type of adult PLC is hepatocellular carcinoma (HCC), which is one of the most common cancers worldwide. It has a higher prevalence in developing countries with increasing number of cases^1^. Liver metastases by other tumor entities are even more common than PLC^2^ and are led by breast cancer metastases. Up to 5.2% of patients with breast cancer develop SLC^3^. In general, liver tumors are difficult to treat. Surgery and local therapies such as radiofrequency ablation (RFA) or microwave ablation (MWA) are possible treatment options. Transarterial chemoembolization (TACE) is an established alternative^4–7^. TACE is treating the tumor by injecting chemotherapy (CTx) medication into the arteries feeding the tumor with additional vessel closing agents to withdraw oxygen and nutrients. Thereby, it promotes the degree of necrosis and apoptosis in the tumor cells^8^. If the tumor decreases in size, it’s a response to the therapy. Therefore, it can be used both as bridging strategy for transplantation or as an effective non-curative palliative treatment approach to PLC^9,10^. In addition, SLC can be downsized for resection or relief of symptoms and improvement of quality of life. Imaging modalities are important for both therapy assessment and follow-up. Magnetic resonance imaging (MRI) and computed tomography (CT) scans are commonly used in daily clinical routine. In addition, magnetic resonance elastography (MRE) represents an innovate imaging technique to quantify TACE efficacy that showed higher accuracy compared to sonography-based elastographic measurements^11,12^. In 2020, Vogl et al. investigated 42 patients with colorectal liver metastases treated with TACE and then examined by MRE. They demonstrated a significant increase of stiffness during the TACE cycles with a parallel decrease in size^12^. Other studies showed a difference of stiffness in cancer cells after different kind of treatments^13–18^. One study in 2017 reported a significant correlation between tumor stiffness, necrosis, and enhancement in HCC after loco-regional therapy (with TACE)^15^. Praktjknjo et al.^19^ demonstrated that a responding HCC after TACE had a significantly higher stiffness compared to non-responding HCC after a short period of time (three days). However, limitations of the above-mentioned studies were small study populations and up to date there has been no direct comparison of PLC and SLC. The aim of our study was to investigate differences in PLC and SLC response to TACE therapy regarding stiffness using MRE as a noninvasive method. Difference in response to TACE could have a major therapeutic impact. Therefore, MRE may provide an added value for evaluation of treatment response in terms of increasing stiffness.

## Material and methods

### Study design and population

From 04/2017 to 10/2017, 58 patients with PLC or SLC underwent TACE at our institution and were included in this retrospective study.

All patients fulfilled our inclusion criteria: age between 18 and 85 years, histopathological and/or radiological evidence of HCC or breast cancer metastases in the liver, current therapy with TACE, and additional MRI and MRE series. Exclusion criteria were liver cancer of different origin, inflammation in the liver or gall bladder, deviations from the standard TACE or MRI/MRE protocol, or exceeding the time limit of 48 h between TACE and MRE acquisitioning.

31 patients suffered from HCC representing the PLC group, whereas 27 women with breast cancer and liver metastases represented the SLC group.

All patients underwent standardized TACE (using mitomycin, gemcitabine and lipidol) with following MRI and MRE of the main tumor (in case of multiple cancer lesions, the largest one was evaluated). For both unifocal and multifocal lesions a (multi-)segmental, in extreme cases a lobar approach was appropriate for treatment. The image acquisitioning occurred directly after the TACE therapy (*n* = 56) or within 48 h (*n* = 2). The maximum time between TACE and imaging lay within a range of 0–2 days. To avoid bias in case of inconsistencies in timing in between TACE and MRE snapshots and varying treatment effects after 48 h, patients exceeding this time limit were excluded. The patients underwent TACE and MRE in 4- to 6-week intervals.

### TACE performance

**Figure 1:**
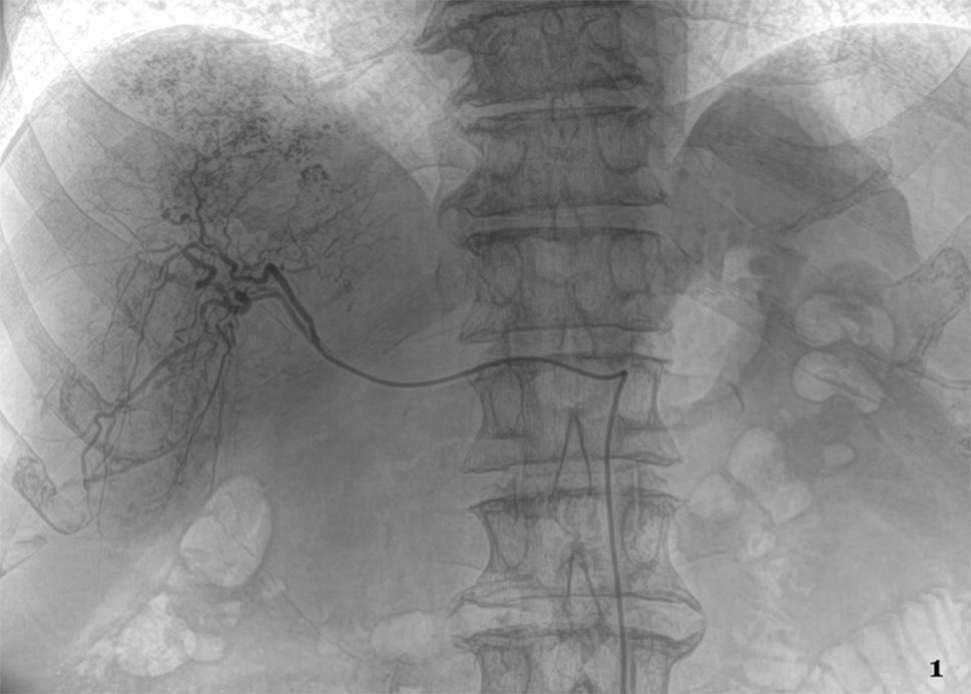
X-ray image. 64 years old male patient with PLC in the RLL during TACE procedure.

In preparation for TACE, a pigtail catheter was inserted via Seldinger technique into the femoral artery^13^. After placing a cobra or sidewind catheter in the celiac and superior mesenteric artery, the tumor feeding vessels were visualized with contrast solution. A preceding MRI was used to identify the correct segmental artery in the liver and small micro-catheters (2.3–3.0 F) were used to prevent vasospasms. After verification of the correct catheter positioning, the cytostatic agents were administered under imaging control [mitomycin C (8 mg/m^2^ body surface area (BSA)), gemcitabine (500 mg/m^2^ BSA), and cisplatin (30 mg/m^2^ BSA)]. For emulsification a suspension consisting of a fixed dosage of Mitomycin C (10 mg) and a maximum dose of 10 ml Lipiodol was injected, which capitation was not correlated with the efficacy of TACE because of the frequently inhomogeneous appearance. At the end of the administration of anticancer-in-oil emulsion 5 ml universal temporary embolic agents such as Embocept® (starch microspheres) were regularly applied to achieve temporary arterial embolization. All TACE interventions were performed using a robot-supported angiography system (Artis pheno, Siemens Healthineers) (see Fig. 1).

### MRI and MRE acquisition

**Figure 2:**
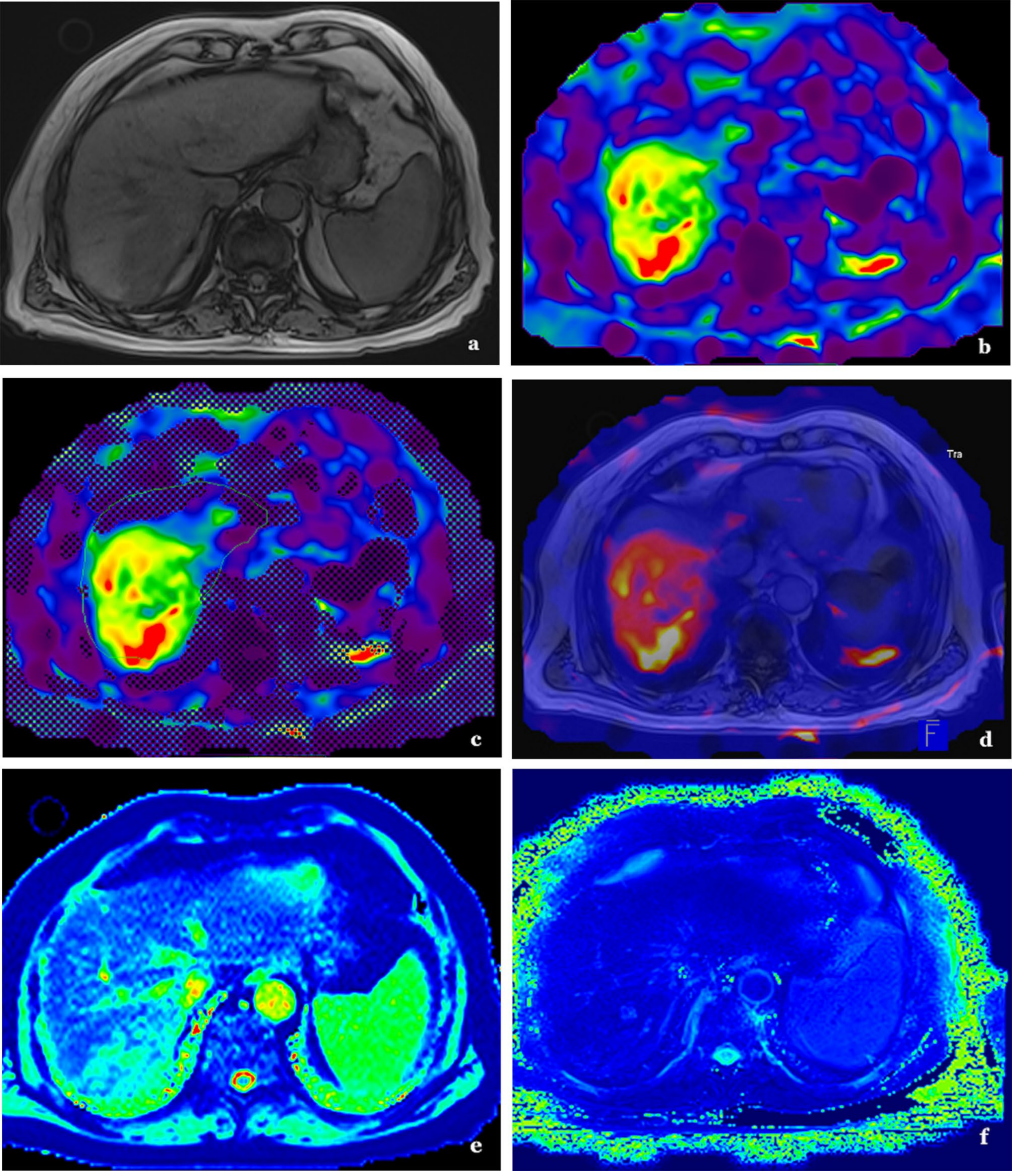
MRI and MRE images. 64 years old male patient with HCC in the RLL with unenhanced T1-imaging (**a**), MRE without (**b**) and with (**c**) liver detection system, 3D-fusion of MRI and MRE (**d**) and MAP T1 (**e**) and MAP T2 (**f**) imaging.

Directly after the intervention, unenhanced and contrast-enhanced MRI was performed using gadobutrol (Gadovist 1 mmol/ml, Bayer Healthcare). There were T1- and T2-weighted MRI and MRE scans acquired in both transverse and sagittal orientation with 5 mm slice thickness using a common 1.5-T system (MAGNETOM Avanto, Siemens). For MRE measurements, a commercially available system was used which consisted of an active driver located outside the scan room connected to passive actuators in the scan room. The time of vibrations was calculated with 15 s for five slices of EPI (WIP measurement) and 23 s for a single slice of GRE, so that a median time of 18 min could be fulfilled for the MRE measurement including patient and system preparation. As previously published, a special sequence protocol was applied^12^. To guarantee high quality images, a vibration frequency of 60 Hz was applied and images were acquired during inspiratory breath hold. In addition, data was collected evaluating magnitude image, phase image, wave image, color-coded elastogram and confidence map (see Fig. 2a-f). The area to which the object resists deformation in response to an applied force (stiffness) was studied in magnitude of the complex shear modulus. This metric could be obtained directly from scanner outputs. MRI data and MRE measurements were matched using a software (Maplt Software, Siemens) ensuring high-resolution segregation of intrahepatic structures and precise measurements of elastography. Evaluation of treatment response was performed with the aid of 3D fusion images including MRI and MRE scan and contained total area of the liver (cm^2^), total liver stiffness (kPa) (including healthy parenchyma and tumor), total liver MAP T1 (ms) and MAP T2 (ms), left and right lobe separated area, stiffness, and MAP T1/T2 measurement, metastasis area, stiffness, and MAP T1/T2. Additionally, measurements in the healthy liver tissue were performed as control.

### Statistical analysis

Data is shown as mean ± standard deviation if normally distributed or as median range if not normally distributed. Continuous variables were tested for normal distribution with the use of the Kolmogorov–Smirnov-test. A Student’s t-test was performed for continuous variables if normally distributed and a Mann–Whitney U test was performed for continuous variables if not normally distributed for comparison between two groups. Categorial variables are given as frequencies and percentages. Spearman´s test was used to assess correlation. Statistical significance was assumed when the null hypothesis could be rejected at *p* < 0.05 and was conducted with SPSS Statistics Version 22.0 (IBM Corporation, Somer, NY).

The investigators initiated the study, had full access to the data, and wrote the manuscript. All authors vouch for the accuracy and completeness of the data and all analyses and confirm that the study was conducted according to the protocol.

### Informed consent

All methods were carried out in accordance with relevant guidelines and regulations. Informed consent was obtained from all subjects and/or their legal guardian(s).

## Results

### Baseline characteristics

Baseline characteristics of the study cohort are shown in Table 1. A total of 58 patients with either PLC (HCC, *n* = 31) or SLC (metastases of breast cancer, *n* = 27) underwent TACE therapy following the same protocol. Patients with PLC were mainly represented by male (*n* = 25, 80.6%), while all patients with SLC were of female gender (*n* = 27, 100%). In the majority of patients, cancer had affected the right liver lobe (RLL) (*n* = 40, 69%). The number of performed TACE cycles before the first MRE acquisition was on average 3.7 (range 0–17) in the PLC group and 3.0 (range 0–15) in the SLC group. Before the first MRE imaging, there were 3.3 TACE interventions performed on average.

**Table 1:** Baseline characteristics.

	All patients (*n* = 58)	PLC (*n* = 31)	SLC (*n* = 27)
Age (years)	65.4	69.6 (39–85)	61.2 (39–81)
Male gender, *n* (%)	25 (43.1)	25 (80.6)	0 (0)
Cancer in right liver lobe, *n* (%)	40 (69.0)	23 (74.2)	17 (63.0)
Number of TACE before first MRE, *n*	3.3	3.7 (0–17)	3.0 (0–15)

### Procedural characteristics

As previously published^12^, the complete procedure including installing the catheter tips, positioning checks and chemoembolization took 35 min in median (data not shown).

### MRE measurements

The results of the MRE measurements are listed in Table 2. The PLC group had a median total liver area of 166 ± 42.9 cm^2^ and a total liver stiffness (corresponding to total liver elastography) of 3.6 ± 1 kPa. The RLL (114.7 ± 36.9 cm^2^) was larger than the left liver lobe (LLL) (54.6 ± 13.8 cm^2^), but the stiffness of the RLL (3.3 ± 1.2 kPa) was comparable to the stiffness of the LLL (3.2 ± 1.4 kPa). The PLC averagely showed a size of 26.4 ± 27.2 cm^2^ and a cancer stiffness of 5.8 ± 1.2 kPa with a MAP T1 of 776 ± 157.4 ms and a MAP T2 of 80.7 ± 26.1 ms. The reference elastography in healthy liver tissue in this group was 2.1 ± 0.5 kPa on average.

**Table 2:** Comparison of results after the first and the second TACE therapy in the group of PLC and SLC.

Parameter	PLC cycle 1	PLC cycle 2	*p*-value	SLC cycle 1	SLC cycle 2	*p*-value
Total liver extent (cm^2^)	166.0 ± 42.9	165.0 ± 50.7	**0.02**	130.0 ± 23.3	134.9 ± 28.0	**0.05**
Total liver elastography (kPa)	3.6 ± 1.0	4.1 ± 1.1	** < 0.001**	2.8 ± 0.7	2.8 ± 0.6	** < 0.0001**
MAP T1 total liver (ms)	686.2 ± 148.8	656.8 ± 53.5	**0.001**	654.0 ± 93.0	648.1 ± 93.1	0.6
MAP T2 total liver (ms)	71.8 ± 13.0	76.3 ± 25.8	**0.03**	64.0 ± 7.0	63.8 ± 8.1	**0.001**
Left lobe extent (cm^2^)	54.6 ± 13.8	52.9 ± 16.2	0.2	36.0 ± 10.6	34.6 ± 10.5	0.8
Left lobe elastography (kPa)	3.2 ± 1.4	3.2 ± 1.3	**0.006**	2.8 ± 1.4	2.5 ± 0.6	** < 0.0001**
MAP T1 left lobe (ms)	679.4 ± 155.3	615.6 ± 64.0	** < 0.001**	656.3 ± 130.1	660.9 ± 90.3	**0.03**
MAP T2 left lobe (ms)	70.2 ± 16.6	70.8 ± 24.5	**0.02**	64.2 ± 10.0	63.2 ± 9.8	**0.001**
Right lobe extent (cm^2^)	114.7 ± 36.9	107.0 ± 23.7	**0.01**	88.5 ± 17.4	92.2 ± 16.0	**0.06**
Right lobe elastography (kPa)	3.3 ± 1.2	3.9 ± 1.5	**0.02**	2.8 ± 0.8	2.8 ± 0.7	**0.01**
MAP T1 right lobe (ms)	686.2 ± 152.2	666.4 ± 56.3	**0.03**	639.2 ± 100.8	651.9 ± 90.4	0.2
MAP T2 right lobe (ms)	73.7 ± 17.1	80.0 ± 27.9	** < 0.0001**	63.1 ± 7.6	63.4 ± 9.0	**0.006**
Cancer extent (cm^2^)	26.4 ± 27.2	21.2 ± 25.2	0.8	11.0 ± 8.7	5.5 ± 5.5	** < 0.0001**
Cancer elastography (kPa)	5.8 ± 1.2	6.9 ± 1.4	**0.002**	5.1 ± 1.4	5.4 ± 1.8	**0.006**
MAP T1 cancer (ms)	776.0 ± 157.4	668.1 ± 92.2	0.3	773.4 ± 243.0	757.7 ± 201.5	**0.02**
MAP T2 cancer (ms)	80.7 ± 26.1	94.2 ± 71.0	** < 0.0001**	73.4 ± 15.9	65.8 ± 21.1	0.9
Reference elastography (kPa)	2.1 ± 0.5	2.4 ± 0.6	0.2	1.8 ± 0.3	1.9 ± 0.4	**0.006**

*kPa* Kilo pascal. Significant values are in bold.

The SLC group had a median total liver area of 130 ± 23.3 cm^2^ and a total liver stiffness of 2.8 ± 0.7 kPa. The RLL (88.5 ± 17.4 cm^2^) was larger than the LLL (36 ± 10.6 cm^2^) and the stiffness of the RLL (2.8 ± 0.8 kPa) was similar to the values measured in the LLL (2.8 ± 1.4 kPa). The SLC averagely showed a size of 11 ± 8.7 cm^2^ and a cancer stiffness of 5.1 ± 1.4 kPa with a MAP T1 of 773.4 ± 243 ms and a MAP T2 of 73.4 ± 15.9 ms. The reference elastography in healthy liver tissue in this group was 1.8 ± 0.3 kPa (see Table 2) on average.

**Figure 3:**
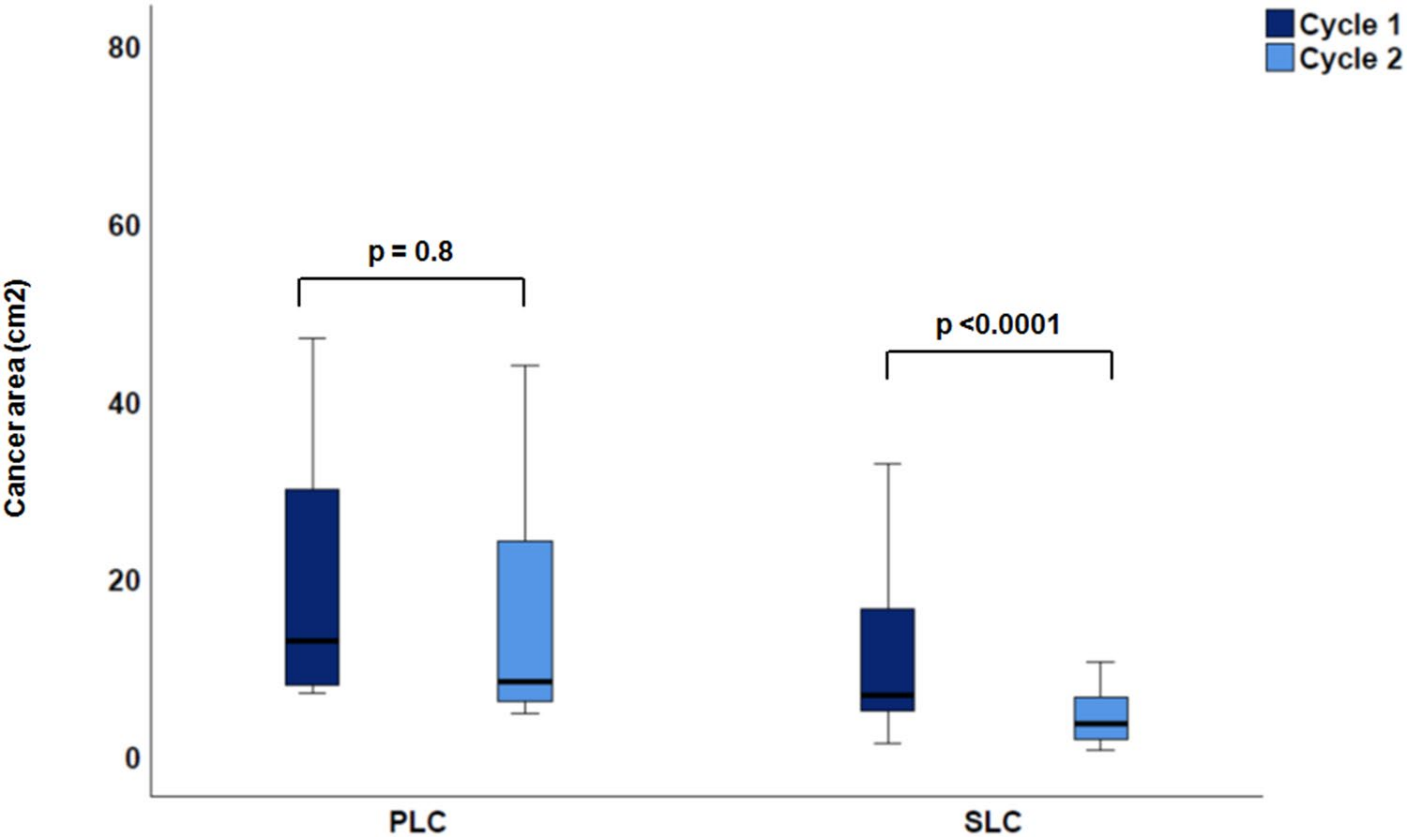
Cancer area in PCL vs. SLC after the first and second cycle of TACE. A decrease of area was shown from the first to the second TACE cycle in each case.

**Figure 4:**
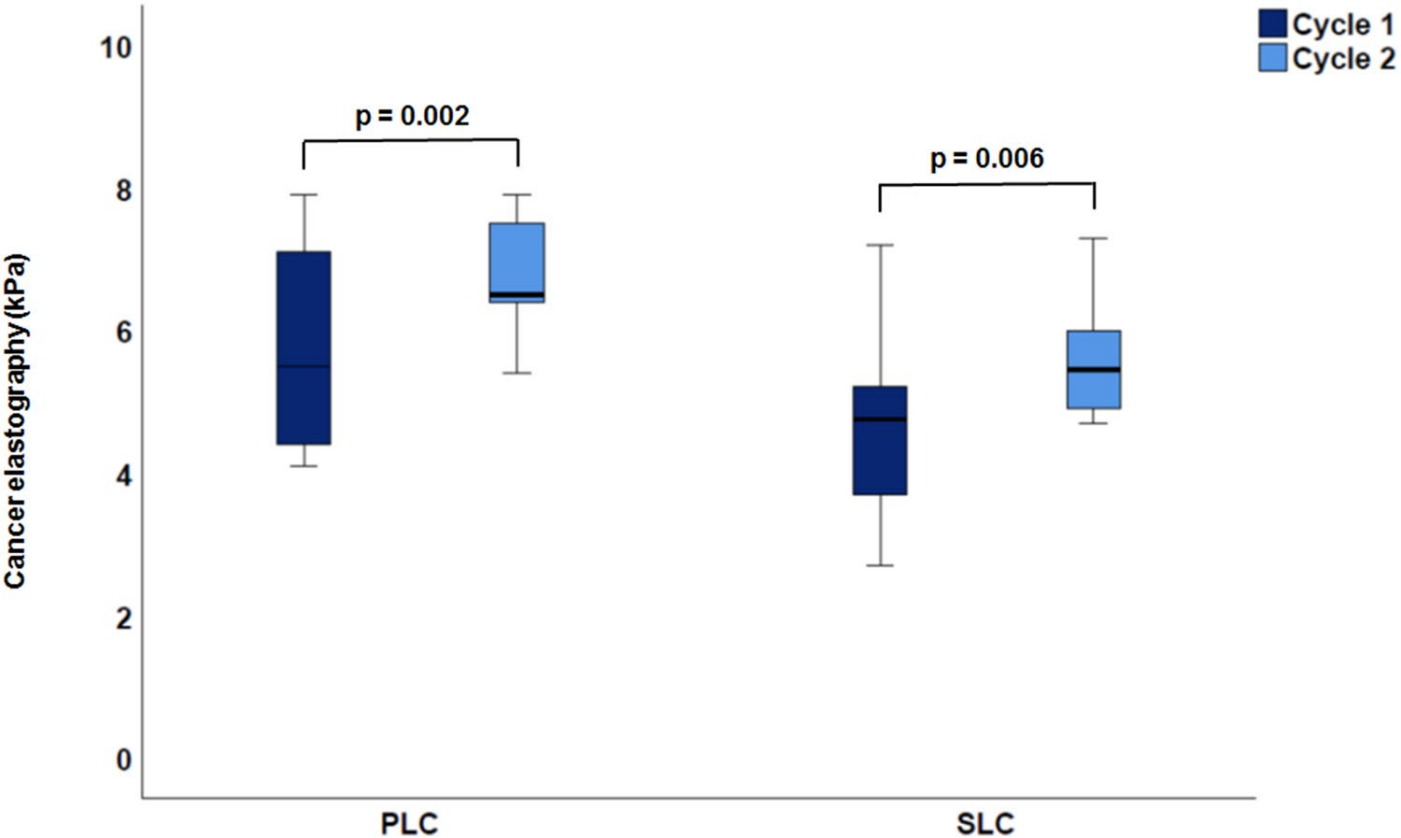
Comparison of elastography in both groups with increasing stiffness from the first to the second cycle (PLC: *p* = 0.002; SLC: *p* = 0.006).

When comparing tumor progression between the first and second TACE cycle in the PLC group (*n* = 9, 29%, see Table 2), total liver area was significantly smaller after the second imaging process (166 ± 42.9 cm^2^ vs. 165 ± 50.7 cm^2^; *p* = 0.02) and total liver elastography significantly increased from 3.6 ± 1 to 4.1 ± 1.1 kPa; *p* < 0.001). Further, total liver MAP T1 and MAP T2 measurements showed significant changes: while MAP T1 decreased (686.2 ± 148.8 ms to 656.8 ± 53.5 ms, *p* = 0.001), MAP T2 increased (71.8 ± 13 ms to 76.3 ± 25.8 ms, *p* = 0.03). From the first to the second TACE cycle, most of the tumorous lesions (*n* = 28, 89%) showed a trend to decrease in size (from 22.3 ± 17.0 to 21.2 ± 25.2 cm^2^, *p* = 0.8) (see Fig. 3), whereas cancer stiffness significantly increased in all lesions from 5.8 ± 1.2 to 6.9 ± 1.4 kPa (*p* = 0.002) (see Fig. 4). The reference stiffness in healthy liver tissue increased from 2.1 ± 0.5 to 2.4 ± 0.6 kPa (*p* = 0.2).

Similar results were detected in the SLC group. Here a total of 18 patients underwent repetitive MRE (67%) (see Table 2). The cancer size significantly decreased from 11 ± 8.7 to 5.5 ± 5.5 cm^2^ (*p* < 0.001) whereas stiffness in tumorous tissue increased from 5.1 ± 1.4 to 5.4 ± 1.8 kPa (*p* = 0.006) (see Table 2, Fig. 3, 4).

**Figure 5:**
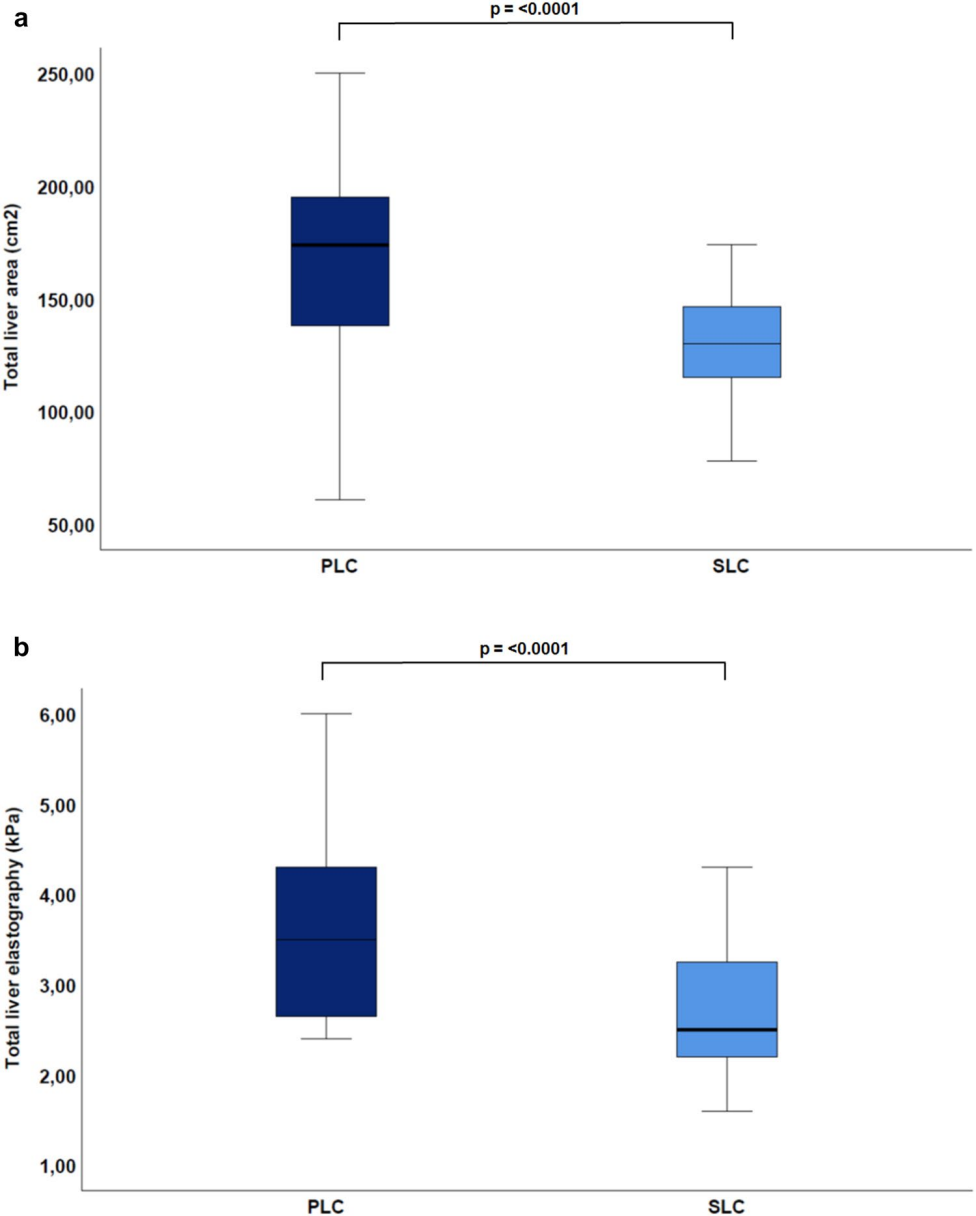
Comparison of PLC vs. SLC total liver (**a**) area (PLC: 166 ± 42.9 cm^2^ vs. SLC: 130 ± 23.3 cm^2^; *p* < 0.0001). (**b**) Elastography (PLC: 3.6 ± 1 kPa vs. SLC: 2.8 ± 0.7 kPa; *p* < 0.0001).

**Figure 6:**
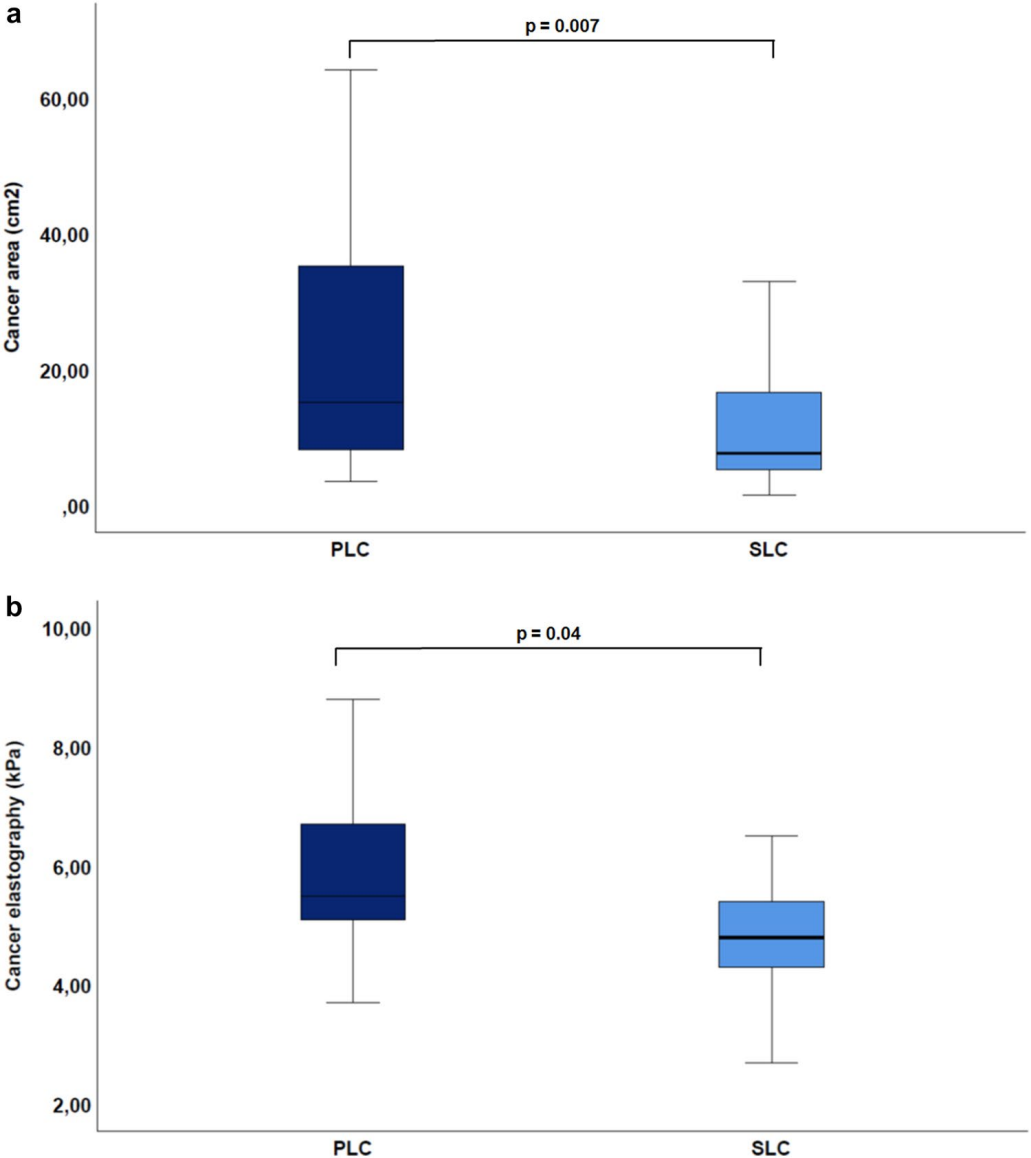
Comparison of PLC vs. SLC (**a**) area (cm^2^) (*p* = 0.007). (**b**) Cancer elastography (*p* = 0.04).

When comparing PLC and SLC measurements, a common increase in cancer stiffness (PLC: *p* = 0.002; SLC: *p* = 0.006) (see Table 3, Fig. 4) after TACE could be detected. The total liver area was greater in the PLC group (166 ± 42.9 cm^2^) than in SLC group (130 ± 23.3 cm^2^; *p* < 0.0001 (Fig. 5a)) and the total liver stiffness was also higher in the PLC group (3.6 ± 1 kPa vs. 2.8 ± 0.7 kPa; *p* < 0.0001 (Fig. 5b)). As a conclusion, stiffness was higher in cancer tissues, regardless of whether these were PLC or SLC, than in the rest of the liver tissue (see Table 3). There was no statistically significant difference in MAP T1 measurement of the whole liver (686.2 ± 148.8 ms vs. 654 ± 93 ms; *p* = 0.40), whereas relevant differences were found in favor of MAP T2 imaging (71.8 ± 13 ms vs. 64 ± 7 ms; *p* = 0.009). With respect to cancer area, the PLC group showed larger tumorous lesions (26.4 ± 27.2 cm^2^) than the SLC group (11 ± 8.7 cm^2^; *p* = 0.007 (Fig. 6a). Moreover, both groups decreased in size from the first to the second imaging (PLC group: 26.4 ± 27.2 cm^2^ to 21.2 ± 25.2 cm^2^, *p* = 0.8; SLC group: 11 ± 8.7 cm^2^ to 5.5 ± 5.5 cm^2^; *p* < 0.001 (Fig. 3)). Concerning cancer stiffness, measured elastography was higher in PLC than in SLC (5.8 ± 1.2 kPa vs. 5.1 ± 1.4 kPa; *p* = 0.04) (Fig. 6b). The reference stiffness in radiologically cancer-free tissue was lower in metastases than in PLC (1.8 ± 0.3 kPa vs. 2.1 ± 0.5 kPa; *p* = 0.06) (data not graphically shown).

**Table 3:** Comparison of PLC and SLC after the first TACE therapy.

Parameter	PLC	SLC	*p*-value
Total liver extent (cm^2^)	166.0 ± 42.9	130. ± 23.3	** < 0.0001**
Total liver elastography (kPa)	3.6 ± 1.0	2.8 ± 0.7	** < 0.001**
MAP T1 total liver (ms)	686.2 ± 148.8	654.0 ± 93.0	0.4
MAP T2 total liver (ms)	71.8 ± 13.0	64.0 ± 7.0	**0.009**
Left lobe extent (cm^2^)	54.6 ± 13.8	36.0 ± 10.6	** < 0.0001**
Left lobe elastography (kPa)	3.2 ± 1.4	2.8 ± 1.4	0.2
MAP T1 left lobe (ms)	679.4 ± 155.3	656.3 ± 130.1	0.6
MAP T2 left lobe (ms)	70.2 ± 16.6	64.2 ± 10.0	0.1
Right lobe extent (cm^2^)	114.7 ± 36.9	88.5 ± 17.4	**0.001**
Right lobe elastography (kPa)	3.3 ± 1.2	2.8 ± 0.8	0.05
MAP T1 right lobe (ms)	686.2 ± 152.2	639.2 ± 100.8	0.2
MAP T2 right lobe (ms)	73.7 ± 17.1	63.1 ± 7.6	**0.006**
Cancer extent (cm^2^)	26.4 ± 27.2	11.0 ± 8.7	**0.007**
Cancer elastography (kPa)	5.8 ± 1.2	5.1 ± 1.4	**0.04**
MAP T1 cancer (ms)	776.0 ± 157.4	773.4 ± 243.0	0.9
MAP t2 cancer (ms)	80.7 ± 26.1	73.4 ± 15.9	0.2
Reference elastography (kPa)	2.1 ± 0.5	1.8 ± 0.3	0.06

*kPa* Kilp pascal. Significant values are in bold.

## Discussion

The present study investigated the effectiveness of MRE as an imaging technique to compare PLC and SLC in addition to conventional MRI and to uncover potential therapy response differences. During therapy, stiffness of tumorous lesions increased from cycle to cycle. This effect was more prominent in PLC. In addition, in most cases a reduction in size was measured. Therefore, MRE may provide an added value for evaluation of treatment response, as an increasing stiffness indicates a response to TACE. In comparison to previous studies^16,19^, we reproduced comparable findings emphasizing the effectiveness and reliability of this innovative imaging technique. It remains unclear why MRE provided better results in PLC than in SLC. A possible explanation could be the volume and composition of the PLC cancer itself, especially its vascular network formation. Accumulation of lipidol in tumorous tissue might lead to a substantial reduction of blood circulation. Furthermore, it is conceivable that a curbed blood supply in turn might result in various modifications of the biomechanical properties, which consequently might affect tissue constitution and ultimately stiffness. As HCCs are very diverse in aggressive behavior, the treatment response might be affected^20^. As shown in a study from Kim, the wide dispersion of stiffness possibly reflects the different biological entities of HCC^21^.

International studies relating to this topic are scarce; there are some studies comparing the degree of stiffness in patients with, inter alia, colorectal liver metastases under CTx with oxaliplatin (OBC, FOLFOX) compared to those without^14,15^. However, it should be noted that the same methodology was not applied to measure stiffness, making comparison difficult. Pelegrina et al.^14^ only involved a well-established ultrasound-based elastography (Fibroscan) to determine liver stiffness; an assessment of liver stiffness by MRE was not performed. A study by Oki et al. took a similar approach using elastography by Fibroscan to evaluate stiffness before and after CTx treatment. Focusing on the short-term outcome, stiffness of tumorous tissue generally increased after CTx within 48 h, and hepatic stiffness was normalized in most cases after 2 weeks^15^. Similar to our results, tumor-free liver elastography was clearly lower, even though a different method was used for measurements. Gordic et al. examined 63 HCC patients with MRE, of which 52 patients underwent Yttrium-90 radioembolization (RE), TACE, or RFA and 11 patients were untreated due to newly diagnosed HCC. After treatment they measured a decrease in cancer stiffness^16^. As opposed to our study, here the decrease in stiffness could be explained by the different therapeutic approaches. Not only liver fibrosis leads to higher liver stiffness^22^, but also necroinflammation that occurs in tumor cells^23–25^. In 2018, Kennedy et al.^22^ published a review about current evidence and future directions in elastography methods in liver disease. Kennedy et al. reported a common trend to an increased stiffness in malignant cells such as HCC. Recently, a higher tumor stiffness was reported in well or moderately differentiated HCCs compared with poorly differentiated HCCs or remaining liver^26^. The pathophysiology to why tumors grow stiffer post-TACE remains unclear. In 2020, Perfahl et al.^27^ reported an unstable tumor state directly after TACE, where regrowth and total tumor death had the same probability. Further, in a study by Motosugi et al.^28^ showed in 2013, liver stiffness in HCC patients was higher than in patients without HCC.

Venkatesh et al.^29^ took a similar approach using MRE to differentiate between malignant (including HCC, cholangiocarcinoma (CCA), and metastases) or benign (including hemangioma, hepatocellular adenoma, and focal nodular hyperplasia) focal liver lesions, whereas we used it to distinguish between PLC and SLC. They were able to demonstrate that malignant focal liver lesions showed significantly higher mean stiffness than benign focal liver lesions. Comparing PLC and SLC, the mean stiffness of HCC was not significantly different from that of CCAs or metastases, establishing a clear trend towards an accentuated increase in stiffness in PLC. Thus their results match what we discovered.

Today, there are various imaging strategies available aiding in the diagnosis and monitoring of treatment success. Fielding et al.^30^ emphasized the importance of MRI, CT and ultrasound in diagnosis and imaging of PLC and SLC, with recommendation for their effective use. While CT is a common imaging technique for HCC screening^31^, ultrasound is a fast and cost effective way of imaging with a great plane resolution, but is dependent on both the quality of ultrasound equipment and experience of the examining physician and very limited in depth^30,32^. The MRI is increasingly used for liver screening with a T1-weighted breath-hold gradient technique as the most sensitive method for HCC detection. The CT angiography is still the preferred method for complete staging of potential tumorous lesion prior to surgery^30^. As common CT and MRI are in black and white film, even when using contrast enhanced agents, elastography measurements are superior in detecting small lesions as they are colored. Also, the MRE measurement took, on average, only 30 min (acquisition of EPI data 15–23 s for 1–5 slices (WIP measurement)) and can be performed on every 1.5-T MR scanner with the use of dedicated hardware and software packages^12^.

Of course, problems in differentiation between PLC and SLC remain due to a high variance in their appearance. Therefore, new imaging techniques are needed to distinguish between the various entities of liver malignancies. MRE may therefore provide an added value for evaluation of treatment response in terms of increasing stiffness. Furthermore, in their review about quantitative elastography methods in liver disease Kennedy et al.^22^ claimed, that more research needs to investigate how study results can be used to enhance healthiness. MRE is one of those promising tools. To avoid limited spatial resolution and coverage of current 2D MRE, nonlinear inversion algorithms paired with 3D MRE may help to improve these issues^17,33,34^.

### Study limitations

Sample size, single-center character and the retrospective design are limitations. For further verification and applicability of our results, larger and prospective studies with a control group are needed. There is a potential selection bias because all included patients of the present study cohort were preselected for the TACE procedure itself without conservative or RE-/RFA-counterparts. Also, patients with PLC and SLC at different tumor stages were included, which may affect the accuracy of the measurements. Apart from this, the potential gender bias (only women in the SLC group) is a further limitation of our study. Finally, our results showed a dispersion in stiffness that possibly reflects the different biological entities of HCC^21^, as stiffness of in vivo biological tissue is very dynamic.

## Conclusion

MRE is a feasible and useful imaging tool to evaluate the response to TACE of PLC and SLC and to allow a differentiation between these entities.

## Abbreviations

abbr: Abbreviation
BSA: Body surface area
CT: Computed tomography
CTx: Chemotherapy
EPI: Echo planar imaging
GRE: Gradient echo
HCC: Hepatocellular carcinoma
kPa: Kilo pascal
LLL: Left lobe of liver
MRE: Magnetic resonance elastography
MRI: Magnetic resonance imaging
MWA: Microwave ablation
PLC: Primary liver cancer
RFA: Radiofrequency ablation
RLL: Right lobe of liver
SLC: Secondary liver cancer
TACE: Transarterial chemoembolization
WIP: Work in progress
